# Comments on Professor Peirce’s Paper, “Pyorrhœa Alveolaris”

**Published:** 1894-04

**Authors:** Albert P. Brubaker

**Affiliations:** Philadelphia


### COMMENTS ON PROFESSOR PEIRCE'S PAPER, "PYORRHŒA ALVEOLARIS."[1]

BY ALBERT P. BRUBAKER, M.D., D.D.S., PHILADELPHIA.

In reply to your request, I send to you a few comments on the view which Dr. Peirce has adopted respecting the causation, pathology, and treatment of pyorrhœa alveolaris. It would be superfluous for me to add any facts or arguments from the field of dental pathology which would corroborate the view that pyorrhœa is but a local expression of the gouty diathesis. I will, therefore, simply present a few facts taken from the field of general medicine which will, perhaps, assist in demonstrating the dependence of pyorrhœa on a constitutional disorder.

At the outset it should be made clear as to what pathological condition is embraced by the term " pyorrhœa alveolaris." According to Dr. Peirce, and with him I fully agree, it is not that pathological condition which, beginning at the gum margins as a simple inflammation, advances towards and involves the pericementum, and even leads to the formation and discharge of pus. This is a purely local condition,—though capable of being aggravated by vitiated systemic states,—established by the deposition of salivary salts, on the removal of which the inflammatory process subsides. This form of pyorrhœa is not to be confounded with that specific inflammation of the pericementum which begins at or near the apical extremity of the root, and which is attended by the formation of pus, the absorption of the alveolar process, and the eventual loss of the teeth. This inflammation is of constitutional origin, established by the deposition from the blood of specific matters which chemical analysis has shown to be calcium and sodium urates and free uric acid. The chemical character of these salts is proof positive that their deposition is indicative of a constitutional or nutritional disorder. Owing to the difficulty experienced in removing this deposit, and owing to the fact that it is as constantly being

---

[1] Read before the Eighth District Dental Society, Buffalo, N. Y., February 26, 1894.

renewed, the inflammation and its concomitants have resisted all local treatment which has been employed.

Now, it is this chronic, persistent, destructive form of pericementitis, which is to be regarded as an expression of the gouty diathesis. This differentiation of the pericemental inflammations into two distinct forms—one local, the other constitutional—must be clearly recognized in order to comprehend the pathology and treatment of the disease under consideration.

That the pericemental membrane the vitality of which has been lowered by mechanical or chemical agencies may become the seat of uratic deposits is in harmony with pathological processes occurring in other portions of the body.

The mucous membrane of the bronchial tubes, the vitality of which has been impaired by various causes, may become the seat of uratic deposits, which will give rise to a persistent inflammation, attended by increased and altered secretion, which resists the customary therapeutic means, and yields only to an anti-gout treatment. The mucous membrane of the pharynx, from the deposition of similar materials, will become the seat of an inflammation which is characteristic. The gouty throat is easily diagnosticated. At times small masses of calcium urate are discharged from the mucous follicles of the mucous membrane. Many other instances might be cited confirmatory of this view. Looking at all the facts, it is readily conceivable that the pericemental membrane is just as susceptible to uratic deposits as any other tissue, and that a gouty pericementitis (otherwise pyorrhœa alveolaris) finds its analogue in gouty bronchitis, gouty pharyngitis, etc.

It is needless to say that in all these instances the patient was the victim of that perverted nutrition which is known by the term gouty.

That this form of pyorrhœa, which Dr. Peirce regards as an evidence of the gouty diathesis, is so in reality is demonstrated by the fact that the deposit is similar in all respects to deposits found in other tissues the seat of inflammations long since recognized as of gouty origin. The objection that patients suffering from this persistent pyorrhœa are not always gouty is only valid when it has been proved that they are not gouty. The burden of proof must rest with the objectors, in view of the facts which have been adduced regarding the causation, and which will be adduced in reference to treatment of pyorrhœa. The common assumption that gout is but an inflammation of the joint of the large toe or the joints of the fingers is highly erroneous. This diathesis may manifest itself in a multiplicity of ways. Nervous disorders, cutaneous inflammations, visceral derangements innumerable may be established by the deposition of uric-acid salts. The diagnosis of these gouty manifestations is only possible with those clinicians who have familiarized themselves with the protean character of this diathesis. It is a fair assumption, and one supported by observation, that the large majority of pyorrhœa patients present other symptoms of gout. A careful examination of the patient's physical condition and an investigation into his family history will frequently, if not always, disclose some gouty manifestation.

In dental medicine, as in general medicine, the specific character of any given pathological condition may frequently be inferred with a reasonable degree of certainty from the results of specific treatment. If some obscure chronic inflammation, which has resisted all forms of medication, should yield and disappear under the administration of quinine, mercury, or colchicum and the alkalies, it would be a fair assumption that the underlying cause of the morbid state was the malarial, syphilitic, or gouty diathesis. If a chronic inflammation of the pericementum, after resisting local treatment for months and years, should yield and disappear under an anti-gout treatment, the practitioner would be justified in regarding the disease as but a manifestation of gout. Now, the facts which have been elicited by Dr. Peirce in the treatment of pyorrhœa absolutely support this view. An outline of the treatment adopted by him has already been published. A detailed treatment with the result is forthcoming. It is only necessary for me to say that it is strictly along the lines of dietetic, hygienic, and medicinal means adopted by clinicians in the treatment of all forms of gout.

---

